# Partial enumeration of extreme rays in metabolic networks using bit pattern trees

**DOI:** 10.3389/fbinf.2026.1892684

**Published:** 2026-09-01

**Authors:** Wannes Mores, Satyajeet Sheetal Bhonsale, Filip Logist, Jan F. M. Van Impe

**Affiliations:** BioTeC+ Chemical & Biochemical Process Technology & Control, KU Leuven Campus Gent, Ghent, Belgium

**Keywords:** constraint-based modelling, elementary flux modes, extreme pathways, extreme rays, metabolic network, tree traversal

## Abstract

Extreme ray analysis of metabolic networks, even though very powerful, is currently limited to smaller metabolic networks. Some approaches to generating partial sets of extreme rays exist, but the computational efficiency of the so-called double-description method is yet to be exploited. Previous work highlighted the possibility of sampling within its iterations, enabling partial enumeration for double-description based methods. However, these approaches severely lack computational efficiency to be a suitable alternative. In this work, the highly efficient bit pattern trees are used within the sampling framework to significantly enhance its output and speed. Combined with the recent revision of the Canonical Basis Approach (CBA), our approach outperforms the other tested methods under the reported benchmark conditions even for a full enumeration study, requiring only half the computation time. In addition, a filter setting allows the memory demand to be scaled down while retaining high efficiency. However, some issues with the combinatorial explosion of candidates still persist and are further investigated. This study therefore puts forward a novel, double description-based alternative to partial enumeration of extreme rays. Further improvements in memory efficiency would allow this promising approach to scale powerful extreme ray-based analyses to genome-scale metabolic networks.

## 1 Introduction

Genome-scale metabolic networks are well established in computational systems biology to study metabolic capabilities and predict behaviours of organisms. For applications such as bioprocesses, where the organism lies at the basis of producing valuable products, prediction of behaviour is key. The information contained in metabolic networks on the microscopic level can inform the process dynamics, leading to multi-scale modelling approaches that enable monitoring and optimization (Pennington et al., 2024; de Oliveira et al., 2021; Vercammen et al., 2016). In addition, the networks can help understand and predict the effects of genetic modifications, leading to optimization on the host level. Usually, metabolic networks are defined through a set of reactions R and a set of metabolites M , with the stoichiometry of each reaction captured in the matrix $S\in R^{\left|M|\times |R\right|}$ . This matrix lies at the basis of the constraint-based modelling approach through the (pseudo-)steady-state assumption (Equation 1):

S⋅v=0 (1)

Where $v\in R^{|R|\times 1}$ is the vector of fluxes through each reaction. The set of irreversible reactions in the system Irr is used to define the feasible solution space or steady-state flux cone (Equation 2):

$$\left\{v\in R^{|R|\times 1}\;|\;S\cdot v=0;\;v_{\mathrm{Irr}}\geq 0\right\}$$ (2)

The analysis of the network *in silico* is important for bioprocesses due to the underdeterminacy of the linear system defined by Equation 1. Even when supplied with a set of measured uptake and secretion fluxes to further constrain the solution space, the linear system is typically not full column rank. This is especially true when dealing with genome-scale networks, where the number of reactions and therefore columns is easily within thousands. The problem of underdeterminacy is thus an important hurdle in many applications of metabolic networks, an overview of this issue along with proposed solutions can be found in Bogaerts and Vande Wouwer (2021).

One of the ways of dealing with the underdeterminacy of the system is through analysis of the solution space. Extreme rays such as Elementary Flux Modes (EFMs) (Schuster et al., 2000) and Extreme Pathways (EPs) (Schilling et al., 2000) characterize the solution space, allowing each feasible solution to be explained through a combination of the extreme rays. They are all the minimal generating set of vectors of a specific (augmented) flux space, but their difference lies in their minimality and whether or not they contain all non-decomposable pathways. A more in-depth investigation into the characteristics of each type can be found in Llaneras and Picó (2010).

Due to their strong capacity of breaking down the complexity of the network, extreme rays have been used for the creation of smaller-scale bioprocess models. This is done through the selection of relevant rays for a given process and taking the overall conversion as a macroscopic bioreaction, which is then dynamically modelled separately afterwards (Maton et al., 2022; Zamorano et al., 2013; Provost and Bastin, 2004). The dynamic macroscopic bioreaction models created are able to explain experimentally observed behaviour well, but required different dynamic models for the different phases (Zamorano et al., 2013) (Hybrid) cybernetic modelling (Ramkrishna and Song, 2018; Song and Ramkrishna, 2011) is a framework that is more robust when growth is not balanced, and therefore does not require decomposition into smaller models. It also relies on extreme rays as the building blocks of the model, but adds the cybernetic variables which deal with the synthesis and activity of enzymes. The formulation of these cybernetic variables rely on a metabolic objective, usually through assuming maximal carbon uptake (Luo et al., 2023; Ramkrishna and Song, 2018).

Other applications of extreme ray analysis include many genetic modification studies, where the extreme rays provide an overview of all possible states of the cell (Harder et al., 2016; Unrean et al., 2010; Schuster et al., 1999). This is used to quantify the effect of genetic modifications on all theoretically possible metabolic states instead of relying on a single flux distribution obtained from an FBA simulation. This leads to a more robust overview of the genetic modification and allows easy analysis of the total effect of the modification on the cell’s metabolism.

Enumeration of the extreme rays for metabolic networks is a computationally challenging task, often requiring long and memory-intensive computation. For most extreme rays, the rays are a direct output of a vertex enumeration problem. Typical vertex enumeration algorithms include the reverse search algorithm and the double description method. Due to its better performance when dealing with highly-degenerate problems typically present in metabolic networks, the double description method is the focus in many previous studies. The canonical basis method (CBA) (Schuster et al., 2000) and the null-space approach (NSA) (Gagneur and Klamt, 2004) are popular adaptations of the double description method, with NSA often considered to be the more performant of the two.

At every iteration, methods based on double-description make combinations within the current set of rays, which leads to the final iterations having large sets of candidate combinations. The most time-consuming step for these types of methods is evaluating each of these pairs to find the elementary candidates among them. By representing the candidates as binary vectors (Gagneur and Klamt, 2004) which indicate reaction participation, they can be seen as k -dimensional tuples and are therefore suitable to more optimized searching methods such as k-dimensional (k-d) trees (Bentley, 1975). By partitioning in a k-dimensional space, data can be organized in a tree structure. In these k-d trees, each node splits the data based on a single dimension until its leaves are reached. Leaf nodes then represent the smallest partition of the data, in many cases individual data points. Traversal of the tree then allows for a very efficient search method in many applications, especially if the nodes split the data on relevant attributes to the search. For example, if the root splits the data evenly on an attribute directly related to the query, half of the data is eliminated instantly.

Terzer and Stelling (2008) adapted the NSA method to include a double tree traversal using a bit-pattern based candidate narrowing step, which led to a much more efficient enumeration method (efmtool). For every iteration of NSA, efmtool organizes large sets of rays in bit pattern trees, which is an adaptation of k-d trees. Bit pattern trees are similar, but instead of splitting a high-dimensional datapoint consisting of continuous data, their internal nodes recursively split themselves based on the binary reaction participation data from Gagneur and Klamt (2004). Which reaction activation is chosen for a node is based on how it splits its current set of rays. The rays are thus stored in the tree as binary bit patterns, since only the absence or presence of the reactions matter. After tree construction, a recursive tree traversal is initiated starting from the roots of each bit pattern tree, as the NSA iterations tries to find elementary combinations of its current rays. The pair of initial root nodes thus represents all candidates and combination of their children further divides the pool of candidates. With the introduction of cut patterns, each combination of children nodes is kept based on whether or not it theoretically could produce an appropriate candidate with any of their leaf nodes. Many combinations of internal nodes will be eliminated this way, thus leading to huge reductions of candidate testing if this happens early in the tree traversal. A large reduction in candidates leads to much more efficient iterations of the NSA method, which is why efmtool is still used frequently for medium-scale metabolic networks.

However, the number of EFMs and EPs explodes with increasing network sizes. The double description-based methods are already memory-intensive, but are intractable for recent genome-scale metabolic networks as the intermediate rays cannot be stored in memory. Therefore, recent work by Buchner and Zanghellini (2021) investigates the use of reverse search algorithms for vertex enumeration. Due to its ability for high levels of parallelization and low memory-consumption, EFMs are able to be generated on high performance computation clusters, but needed hundreds of threads in parallel to achieve similar levels of performance to double description-based efmtool.

An alternative way to deal with the exploding sizes of intermediate rays is to sample in the iterations of the double description methods. In Machado et al. (2012), a sampling approach is used to generate subsets of EFMs in a small-scale network. The filter setting K is adapted to return different sizes of EFM sets, with good sampling quality. However, computational efficiency is low due to the necessity of the rank-based elementarity test instead of the more efficient combinatorial test used frequently in efmtool. Recently, Mores et al. (2025) defined a positive-definite elementarity test for CBA, which significantly improves the efficiency of CBA for sampling-based approaches. Implementations of NSA and CBA reveal much faster evaluation of candidates for CBA using the new elementarity test. However, CBA returns fewer rays for the same filter setting, which suggests that CBA is less efficient per candidate, needing more intermediate candidates to be processed to get a similar output. It is this aspect of CBA where k-d trees can provide significant improvement. With better search techniques, the efficiency downside of CBA can be mitigated.

In this work, k-d trees are exploited to make the CBA more efficient. We adapt bit pattern trees to sampling-based approaches to generate extreme rays. Our novel approach leads to more efficient generation by:

- leveraging CBA’s faster elementarity testing,
- exploiting candidate narrowing through bit pattern trees, mitigating CBA’s efficiency issues,
- enabling the scaling of the extreme ray generation problem through a filter in the double tree traversal.

By combining the new elementarity test described in Mores et al. (2025) with the efficiency of bit pattern trees, this work enables a more efficient partial extreme ray enumeration of metabolic networks without the need of high performance computing clusters.

## 2 Materials and methods

### 2.1 Extreme pathways

As discussed in Llaneras and Picó (2010), different augmentations of the stoichiometric matrix lead to different types of extreme rays of the steady-state flux cone. In this work, the focus lies on the generation of Extreme Pathways, but all principles equally apply to other types of extreme rays. To obtain EPs, augmentation of the stoichiometric matrix consists of adding the reversed version of all intracellular, reversible reactions. These reactions are represented through the set $R_{\mathrm{int,rev}}$ , which is used to define the subset of S corresponding to the reversed reactions $-\left.S_{\left[*,\;R_{\mathrm{int,rev}}\right]}\right]$ . Now, the augmented stoichiometric matrix $S^{\prime }$ is defined as a horizontal concatenation of two matrices (Equation 3):

$$S^{\prime }=\left[S\;|\;-S_{\left[*,\;R_{\mathrm{int,rev}}\right]}\right]$$ (3)

The number of columns in the augmented stoichiometric matrix is therefore larger, and has shape $S^{\prime }\in R^{\left|M|\times |R|+|R_{\mathrm{int,rev}}\right|}$ . The enumeration of EPs then boils down to finding all minimal or non-decomposable solutions to the adapted constrained problem (Equations 4–6).

$$S^{\prime }\cdot v^{\prime }=0$$ (4)

$$v^{\prime }\geq 0$$ (5)

$$v^{\prime }\neq 0$$ (6)

Where $v^{\prime }$ is the augmented version of the flux vector v , with additional entries corresponding to the added reverse version of the reactions in $R_{\mathrm{int,rev}}$ . For the shape of the augmented linear system, the notation $S^{\prime }\in R^{\left|M|\times |R^{\prime }\right|}$ and $v^{\prime }\in R^{|R^{\prime }|\times 1}$ is used.

### 2.2 Sampling-based Canonical Basis Approach

CBA is currently the less popular double description-based algorithm for generating extreme rays in metabolic networks. This is due to NSA’s high efficiency in generating the full set of extreme rays, employing a highly efficient combinatorial elementarity test. However, when using sampling approaches, these types of elementarity tests are invalid. Alternative testing includes the computationally intensive nullity test, with a more efficient test recently defined specifically for sampling-based CBA (Mores et al., 2025). That novel elementarity test makes CBA more preferable in sampling-based approaches due to its lower computational burden per test. A downside is the higher number of candidate evaluations needed for CBA to generate the same set of extreme rays. The advantage created by a faster elementarity test is therefore counteracted significantly. As a result, CBA would greatly benefit from decreasing the number of intermediate candidates. A lower number of intermediate candidates would mean a lower number of elementarity test, thus increasing the elementarity testing efficiency of CBA. Therefore, in this work, the sampling-based CBA has been extended with a candidate narrowing step based on bit pattern trees, leading to a novel approach outlined in Algorithm 1. A major difference between this work and the algorithm description in Mores et al. (2025) is that sampling and elementarity testing is combined with the candidate narrowing step. A more in-depth explanation of how this step happens is shown in Section 2.4. The roots of each tree are the sets $T^{+}$ and $T^{-}$ , which correspond to the sets where the current metabolite to be processed is positive or negative respectively.

Algorithm 1 Sampling-based CBA.

**Input:** Augmented Stoichiometric matrix $S^{\prime }\in R^{\left|M|\times |R^{\prime }\right|}$ , filter setting K

**Output:** Matrix containing a subset of EPs of the network
 1: Combine matrices to initialize Tableau $T=\left[I_{\left|R^{\prime }\right|}\;{\left(S^{\prime }\right)}^{⊤}\right]$

 2: **for** i∈1…|M| **do**
 3: p= current number of rows in T

 4: $T^{0}=\left\{x\in 0,1,\ldots ,p:T_{\left[x,\;|R^{\prime }|+i\right]}=0\right\}$

 5: $T^{+}=\left\{x\in 0,1,\ldots ,p:T_{\left[x,\;|R^{\prime }|+i\right]}>0\right\}$

 6: $T^{-}=\left\{x\in 0,1,\ldots ,p:T_{\left[x,\;|R^{\prime }|+i\right]}<0\right\}$

 7:  Initialize new Tableau $T_{\mathrm{new}}=T_{\left[T^{0}\right]}$

 8:  Candidate narrowing $C=\text{Traverse}\left(T^{+},T^{-},K,T\right)$ (Algorithm 4)
 9:  **for** $\left(j^{+},j^{-}\right)\in C$ **do**
 10: $c=T_{\left[j^{-},|R^{\prime }|+i\right]}\cdot T_{\left[j^{+},:\right]}+T_{\left[j^{+},|R^{\prime }|+i\right]}\cdot T_{\left[j^{-},:\right]}$

 11:   Add c to $T_{\mathrm{new}}$

 12:  **end for**
 13: $T=T_{\mathrm{new}}$

 14: **end for**

### 2.3 Bit tree construction

In contrast to the algorithm described in Terzer and Stelling (2008), the bit tree is not fully constructed before the traversal since many nodes will not be traversed as they are not chosen in sampling. Instead, children nodes will be created when they are selected for further traversal.

The splitting is done based on whether or not each candidate has a zero or non-zero entry for a specific bit. This bit is chosen so that the split is closest to a 50–50 split. Then, two children are created, with the left child containing all non-zero entries and the right child containing the zero entries. This novel adaptation of bit pattern tree construction is shown in Algorithm 2.

Algorithm 2 Create child nodes.

**Input:** Tree node T , current Tableau T

**Output:** Children nodes $T_{\leftarrow }$ and $T_{\to }$

 1: **for** $i\in 1\ldots |R^{\prime }|$ **do**
 2: $T_{nz}=\left\{k\in T:T_{\left[k,i\right]}\neq 0\right\}$

 3:  calculate split $s_{i}=\frac{\left|T_{nz}\right|}{\left|T\right|}$

 4: **end for**
 5: Get best 50–50 bit $b=\underset{i\in \left\{1,\ldots ,|R^{\prime }|\right\}}{arg\;min}\|s_{i}-0.5{\|}_{2}$

 6: $T_{\leftarrow }=\left\{k\in T:T_{\left[k,b\right]}\neq 0\right\}$

 7: $T_{\to }=\left\{k\in T:T_{\left[k,b\right]}=0\right\}$

### 2.4 Tree traversal

**FIGURE 1:**
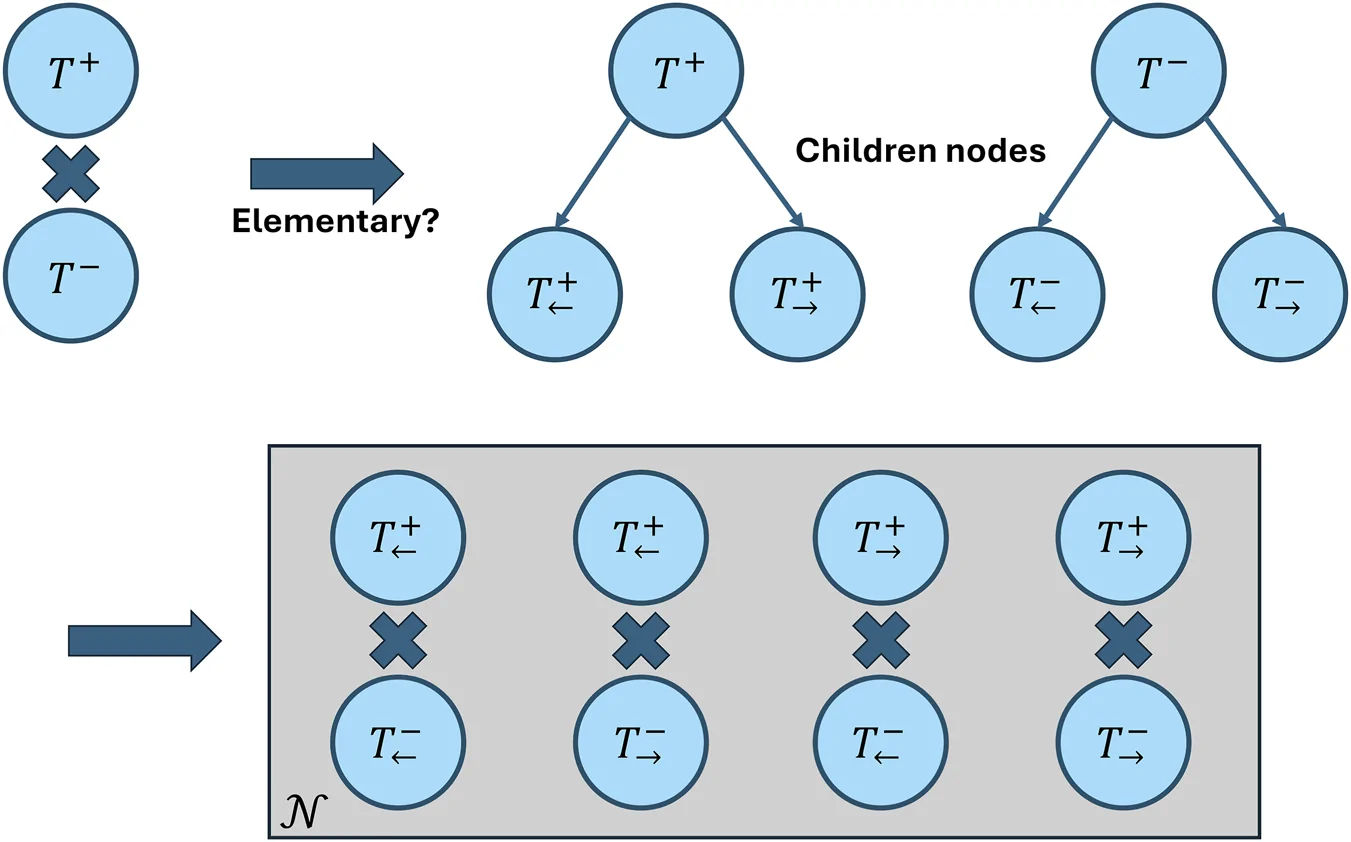
Example of the creation of new nodepairs if a pair of tree nodes passes the elementarity test. First, the new children nodes are created if possible, then, new combinations are made accordingly. This visualisation is specific for the case $|T^{+}|>1$ and $|T^{-}|>1$ (Algorithm 3).

To complete the candidate narrowing step, the trees have to be traversed and checked for elementarity. However, compared to a normal tree traversal, the candidate narrowing step happens by traversing two trees simultaneously. Instead of evaluating nodes separately, pairs consisting of one node from each tree are used. If they are considered to be able to generate an elementary candidate, the nodes from the pairs are split into children nodes and new combinations are made. A visual example of this can be found in Figure 1. For a nodepair with two children nodes each, four new combinations are made. If one of the nodes cannot be split into children, the node itself will be combined with the children of the other node in the pair, leading to two new pairs. For some pairs, when $|T^{+}|=1$ and $|T^{-}|=1$ , the nodepair cannot be split anymore. In that case, the pair represents one single candidate that has passed the candidate narrowing step. It is then added to the array of filtered candidates C . A total overview of the nodepair creation can be found in Algorithm 3, which is inspired by the approach outlined in Terzer and Stelling (2008).

Algorithm 3 Add new nodepairs.

**Input:** Tree nodepair $\left(T^{+},T^{-}\right)$ , current Tableau T

**Output:** Set of new nodepairs N

 1: Initialize set of new nodepairs N={}

 2: **if** $|T^{+}|>1$ and $|T^{-}|>1$ **then**
 3: $T_{\leftarrow }^{+},\;T_{\to }^{+}=\text{create children}\left(T^{+},T\right)$ (Algorithm 2)
 4: $T_{\leftarrow }^{-},\;T_{\to }^{-}=\text{create children}\left(T^{-},T\right)$ (Algorithm 2)
 5: $N=N\cup \left\{\left(T_{\leftarrow }^{+},T_{\leftarrow }^{-}\right),\left(T_{\leftarrow }^{+},T_{\to }^{-}\right),\left(T_{\to }^{+},T_{\leftarrow }^{-}\right),\left(T_{\to }^{+},T_{\to }^{-}\right)\right\}$

 6: **end if**
 7: **if** $|T^{+}|>1$ and $|T^{-}|=1$ **then**
 8: $T_{\leftarrow }^{+},\;T_{\to }^{+}=\text{create children}\left(T^{+},T\right)$ (Algorithm 2)
 9: $N=N\cup \left\{\left(T_{\leftarrow }^{+},T^{-}\right),\left(T_{\to }^{+},T^{-}\right)\right\}$

 10: **end if**
 11: **if** $|T^{+}|=1$ and $|T^{-}|>1$ **then**
 12: $T_{\leftarrow }^{-},\;T_{\to }^{-}=\text{create children}\left(T^{-},T\right)$ (Algorithm 2)
 13: $N=N\cup \left\{\left(T^{+},T_{\leftarrow }^{-}\right),\left(T^{+},T_{\to }^{-}\right)\right\}$

 14: **end if**

The total traversal thus starts from the initial nodepair of the roots $T_{0}^{+}$ and $T_{0}^{-}$ , continuing as long as there are nodepairs to process in N . For each of these nodepairs in N that pass the filter, individual union patterns are created. Their intersection then leads to the cut pattern $U_{\cap }$ , which is used to determine whether the pair can theoretically create an elementary candidate with its leaf nodes. The positive definite elementarity test from Mores et al. (2025) is used to determine the elementarity of this cut pattern.

If true, then children are constructed and new nodepairs are added. For each new depth level of the traversal, the probability of passing for each node is updated based on the size of current nodepairs |N| and filter setting K . This novel adaptation of the double tree traversal with filtering is shown in Algorithm 4.

**FIGURE 2:**
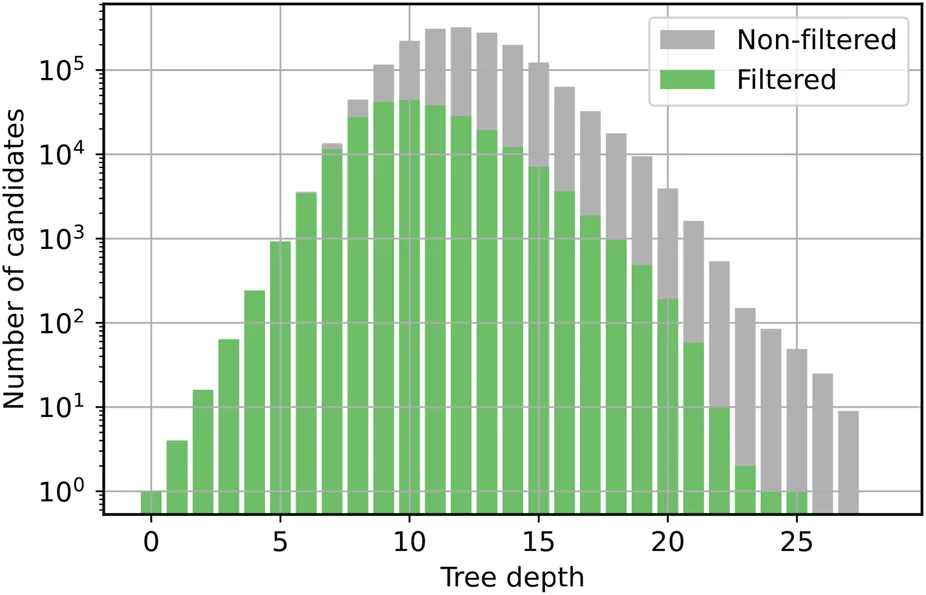
Effect of a filter setting of $K=10^{5}$ on a tree traversal. The number of candidates at each depth level remains the same at the start, with filtering taking place whenever the number of candidates comes closer to K . Afterwards, the number of candidates is consistently lower since the trees are smaller due to the filter.

During the traversal, the set of final candidates will grow. At the end of the traversal, this set is returned as the candidates that passed the candidate narrowing step. The effect of the filter setting is shown in Figure 2, where the same traversal is done with and without filter. K is then a filtering parameter that controls the expected number of node pairs retained at each traversal depth.

Algorithm 4 Tree traversal.

**Input:** Tree roots $T_{0}^{+}$ and $T_{0}^{-}$ , filter setting K , current Tableau T

**Output:** Set of filtered, elementary candidates C

 1: Initialize set of nodepairs $N=\left\{\left(T_{0}^{+},T_{0}^{-}\right)\right\}$

 2: Initialize set of filtered, elementary candidates C={}

 3: **while** |N|≠0 **do**
 4:  initialize next set of nodes $N_{i}=\left\{\right\}$

 5:  Calculate probability $P=\frac{K}{|N|+K}$

 6:  **for** $\left(T^{+},T^{-}\right)\in N$ **do**
 7:   **if** uniform sample X∼U(0,1)≤P **then**
 8: $U_{+}=\text{union pattern}\left(T^{+}\right)$

 9: $U_{-}=\text{union pattern}\left(T^{-}\right)$

 10: $U_{\cap }=U_{+}\cap U_{-}$

 11:    **if** $U_{\cap }$ is elementary **then** (Equation 8)
 12:     **if** $|T^{+}|=1$ and $|T^{-}|=1$ **then**
 13: $C=C\cup \left(T^{+},T^{-}\right)$

 14:     **else**
 15: $N_{\mathrm{new}}=$ Add new nodepairs $\left(\left(T^{+},T^{-}\right),\;T\right)$ (Algorithm 3)
 16: $N_{i}=N_{i}\cup N_{\mathrm{new}}$

 17:     **end if**
 18:    **end if**
 19:   **end if**
 20:  **end for**
 21: $N=N_{i}$

 22: **end while**

### 2.5 Candidate evaluation

As mentioned, each candidate nodepair that has passed the filter is checked for elementarity through its cut pattern $U_{\cap }$ which is obtained by taking the intersection of the union pattern of each node in the pair. The union pattern for a tree node T can be defined as the union of all its zero sets as can be seen in Equation 7 (Terzer and Stelling, 2008).

$$U_{T}=\underset{i\in T}{⋃}Z_{i}$$ (7)

where $Z_{i}$ is the zero set which contains the indices where the i th binary vector in T is zero.

In CBA, the elementarity can be checked efficiently using the positive-definite test. For this test, an extended stoichiometric matrix $S^{*}$ is required, which is obtained by adding a row of ones to $S^{\prime }$ . Its Gramian form then leads to a smaller, square matrix whose positive definiteness indicates elementarity. At iteration k , a candidate nodepair is elementary if the following Equation 8 holds (Mores et al., 2025):

$${\left(S_{\left[1\ldots k,\bar{U_{\cap }}\right]}^{*}\right)}^{⊤}\cdot \left(S_{\left[1\ldots k,\bar{U_{\cap }}\right]}^{*}\right)≻0$$ (8)

The advantage of this test is that positive definiteness is evaluated faster than the typical matrix rank calculations needed for the nullity test in NSA. In addition, even though the Gramian matrix requires a matrix multiplication, it is often significantly smaller than $S^{\prime }$ .

### 2.6 Case studies

To investigate the proposed approach, two case studies are considered based on two well-studied metabolic networks. The e_coli_core model (Orth et al., 2010) is a well-established medium-scale network and serves as the benchmark versus other existing approaches. This is done for its ability for comparison with full enumeration approaches such as efmtool, while still being a network of decent complexity (72 metabolites, 95 reactions). The filter setting K is varied, which showcases its ability to scale the enumeration problem and provide computational efficiency metrics across different problem sizes. For each filter setting, 10 runs are carried out to account for the stochasticity of the method. The variability per filter setting is shown through its median and interquartile ranges.

The genome-scale metabolic network iJR904 serves as the second case study, investigating the applicability of the proposed approach to larger networks ( >1,000 reactions). Again, K is varied until memory limitations are hit. All simulations were executed on a desktop computer with an Intel Core i7-7,700 processor (4 physical cores, eight hardware threads) and 16 GB of RAM. Parallelized tasks were managed using the Python multiprocessing package with default settings, spawning eight independent worker processes to utilize all available logical threads. For efmtool, the same eight workers are made available for fair comparison. Both the networks are compressed using the compression functions from the efmtool package, meaning that each method has the exact same compression applied. The tree-based CBA method proposed in this work is publicly available on Gitlab (https://gitlab.kuleuven.be/biotec-plus/epgenerator). The implementations for both the proposed method and the existing methods such as efmtool are run only on the CPU with multiprocessing enabled over the eight hardware threads. Version 4.7.1 of efmtool is used, with the rank-based test and the MostZeros row ordering option.

## 3 Results

### 3.1 Medium-scale network

**FIGURE 3:**
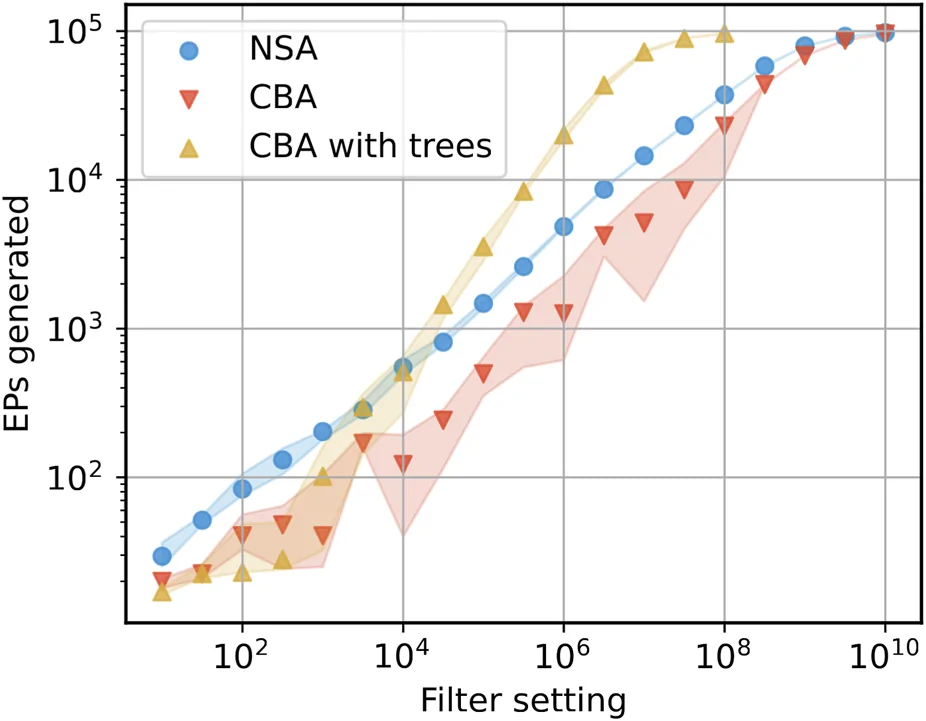
Evolution of number of EPs generated for the e_coli_core model using different filter settings K . Every filter setting is run 10 times to account for the inherent stochasticity of the methods. The median number of EPs generated per filter setting is shown along with the interquartile range as shaded area. Three methods are implemented here, showcasing their ability to scale the extreme ray enumeration problem.

**FIGURE 4:**
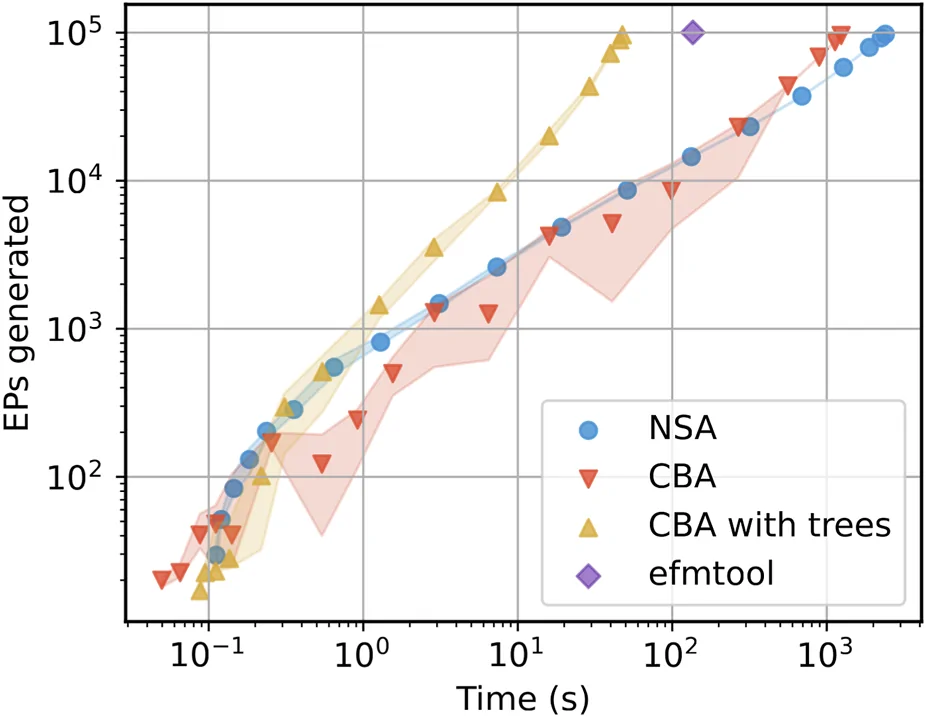
Median computation times versus EPs generated for the e_coli_core model results. Interquartile ranges are shown for the number of EPs generated as shaded area. For efmtool, only the full enumeration is possible, resulting in only one datapoint. Overall, the proposed approach outperformed the other methods under the reported benchmark conditions.

To fully investigate the scaling of the method with different filter settings K , the e_coli_core network is used. The total number of EPs for this network is 100,273, which is used as a stopping criterion. Once this number of EPs is obtained consistently, the filter setting K is no longer increased. As established previously, CBA can perform similarly to NSA in EPs generated for the same amount of time (Mores et al., 2025). For the same filter setting, CBA is usually faster, but returns fewer EPs. In Figure 3, the higher return of EPs of NSA can be observed when compared to CBA without using the tree-based candidate narrowing step. Figure 4 then confirms the previously established observation that NSA and CBA perform similarly in terms of EP return for a specific computation time.

For both CBA-based algorithms there is a higher effect of the stochasticity of the sampling approach as can be seen with the larger interquartile ranges. For higher filter settings, the candidate narrowing step seems to reduce this effect slightly. However, for lower filter settings $\left(10^{1}-10^{3}\right)$ a consistent small set of EPs is generated frequently, leading to large interquartile ranges and a relatively flat section for both CBA-based methods in Figure 4. Most of them are short EPs that might not connect substrate to products and consist mainly of internal loops, which in many applications do not provide useful information. These pathways are known as the type II and type III EPs (Price et al., 2002), which are significantly shorter than the more interesting type I EPs. Further investigation into pathway lengths (see Supplementary Material) showed that these lower filter settings are biased towards shorter pathways, which is common for filtering-based approaches (Mores et al., 2025; Machado et al., 2012). When considering the results of CBA with the candidate narrowing step, the different usage of K must be taken into account. For the same filter setting, the same K value will lead to much more elementarity tests being carried out. For example, in Figure 2, the total number of elementarity tests carried out is around 240, 000. This is significantly higher than the originally defined $K=10^{5}$ . Compared to the non-tree algorithms, a much steeper slope is present, which is partially due to the higher number of tests allowed in the tree traversal. To more fairly compare the algorithms, the number of EPs generated versus computation time should be investigated. This removes the bias introduced due to the different usage of K for the candidate narrowing approach.

Similar to NSA, the tree-based candidate narrowing step vastly improves the efficiency and speed of CBA. Figure 4 shows that the incorporation of the tree traversal increases the enumeration speed of the full set by more than 30 times. Around the 50 s mark, the same computation time leads to 10 times more EPs generated. The full set was also calculated with efmtool, serving as a comparison with a tree-based NSA implementation. The final set of EPs generated by efmtool is identical, but it can be seen that the proposed approach in this work is faster, needing only half the computation time. Efmtool even uses speed improvements through the rank updating method and larger leaf node sizes, which is possible due to its goal of generating all EFMs instead of sampling subsets.

The effect of stochasticity is also present in Figure 4, showing large interquartile ranges for both the CBA-based methods. With increasing computation times and thus larger EP sets for the tree-based CBA, the stochasticity effect diminishes. Based on these results, K shows the ability to consistently scale the problem across the entire range of possible EP set sizes. Our approach therefore achieves its goal of a higher efficiency and computation speed, while retaining control over the resulting EP set size through the filter setting K .

### 3.2 Genome-scale network

**FIGURE 5:**
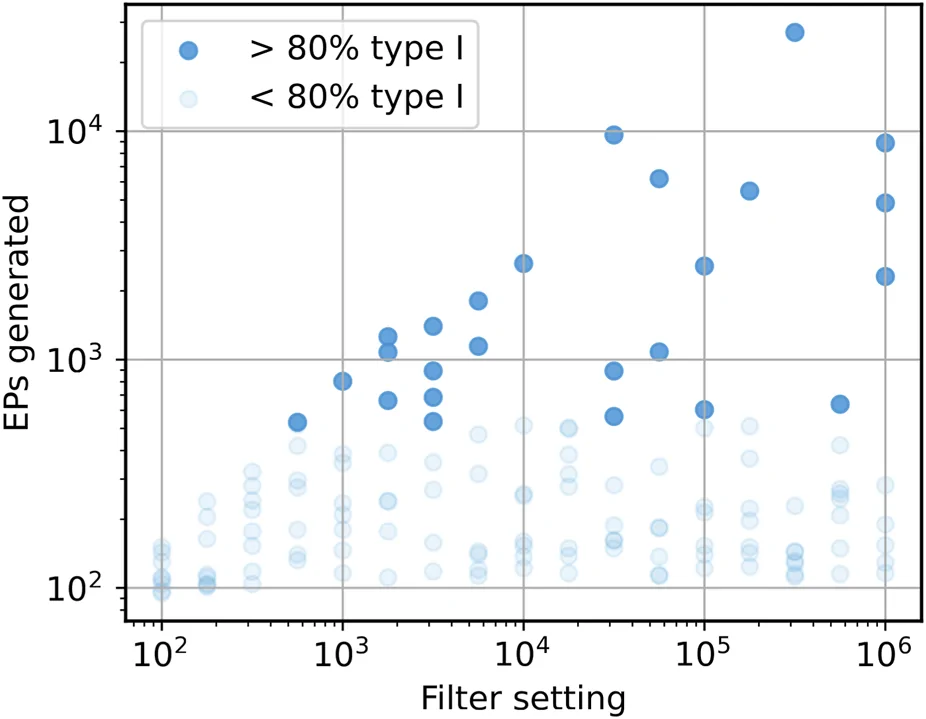
Evolution of number of EPs generated for the iJR904 genome-scale network model using different filter settings K . Every filter setting was used 8 times as many runs resulted in mainly Type II and III EPs, which are shown as faded. The ability to scale the problem with K is investigated based on the solid, mainly Type I runs (>80%) .

**FIGURE 6:**
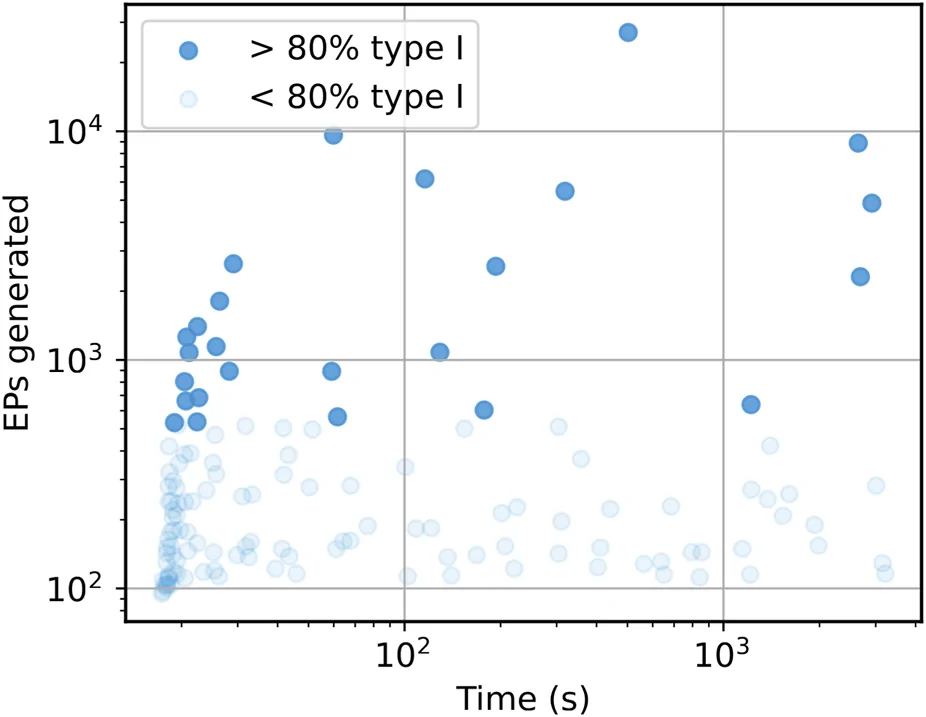
Computation times versus EPs generated for the iJR904 genome-scale network model results. The faded points correspond to runs where mainly Type II and III EPs were generated.

In the second case study, the applicability of the method to a genome-scale network is tested. For these large networks, the full set cannot be enumerated. The filtering approach introduced in this work allows the enumeration problem to be scaled down to reasonable levels of memory usage through its filter setting K . Here, the highest K investigated was $10^{6}$ as memory usage peaked over 16 GB for higher filter settings. The advantage CBA creates with its more efficient elementarity testing is even larger for genome-scale networks. For the interested reader, we refer to Mores et al. (2025), where a more in-depth investigation is done into this phenomenon. In Figures 5–6, the EP output is shown versus the filter setting and computation time respectively.

The results of the genome-scale network show high stochasticity, with large differences in EP outputs for the same filter setting across the entire range. Upon further investigation, the smaller EP sets show much larger proportions of Type II and III pathways. In the results of the previous case study, a bias towards these types of pathways was already confirmed for smaller filter settings. However, in this case study, this effect is seen for all the filter settings tested. When considering only the runs where >80% of pathways generated are of Type I, the scaling of the problem through K is more visible. For higher filter settings, the EP output diverges significantly, with the high Type I runs showing significantly larger returns compared to the Type II and III runs. In the Supplementary Material, more detailed info can be found regarding pathway types and their dominance.

**FIGURE 7:**
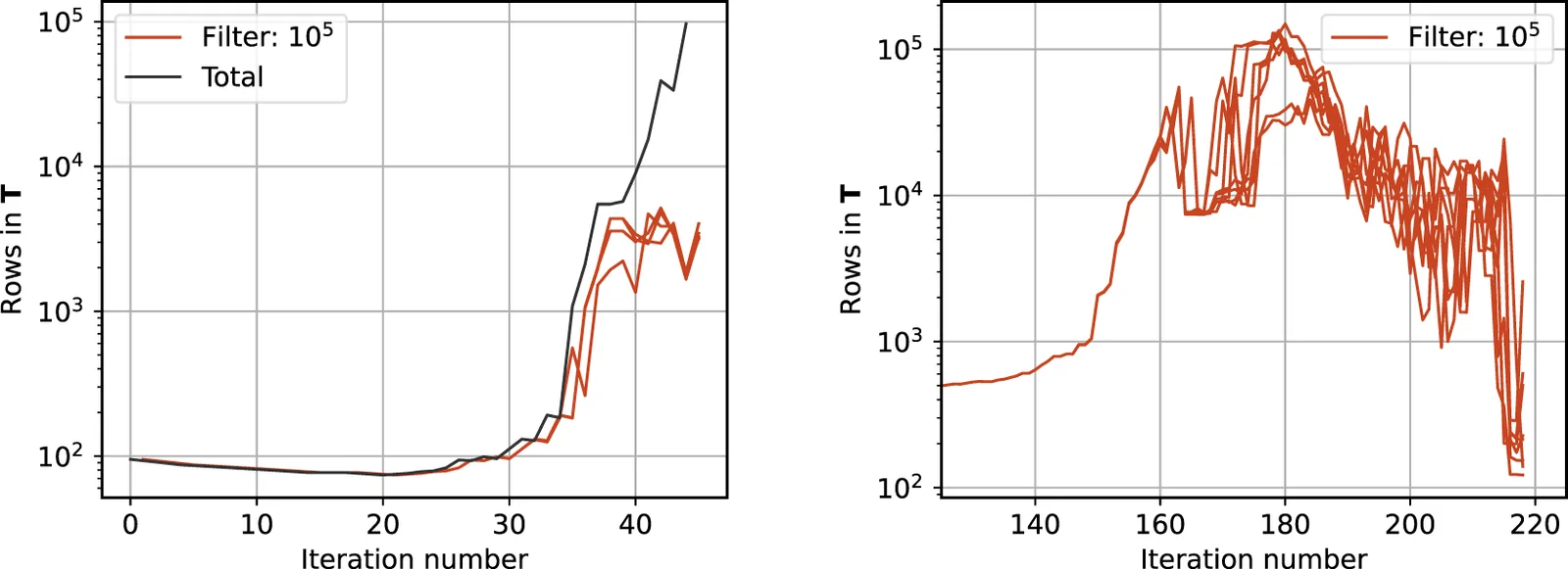
Evolution of number of rows in the tableau throughout the iterations of the tree-based CBA method for the two case studies. The e_coli_core case study (left) shows the combinatorial explosion, with the effect of the filter in later iterations of the method. In contrast, the GEM case study (right) showcases both an explosion and a collapse of candidates in the later iterations of the method. This severely limits the direct impact of the filter setting K on the final output of extreme rays.

As mentioned, K could not be set higher than $10^{6}$ as the RAM usage would exceed 16 GB. This was unexpected since the EP output was not high enough to result in high RAM usage, even for the largely Type I runs. Therefore, the size of T throughout the iterations of the algorithm is investigated. For comparison, this was also performed on the medium-scale network of case study I, which showed steady growth with the last iterations containing the largest number of rows in T (Figure 7). Lower values for K flatten out this curve by filtering out many of these candidates, remaining relatively stable until the final set of EPs. This evolution can be attributed to the ordering used in most algorithms. Iterations with high amounts of candidates are pushed back, while iterations with the smaller candidate sets are processed first. Different ordering methods exist, an overview can be found in Terzer (2009).

However, the evolution of rows in T for the genome-scale network shows a rise of candidates until the filter activates, followed by a large reduction until the final iteration. Especially in the last 10 iterations, where a reduction of around 100 times often occurs (Figure 7). Compared to the expected levelling out, this is a big contrast. Runs where mostly Type II and III EPs are found suddenly lose their candidates in the final few iterations, which could be due to important connections being broken in an earlier iteration. As a result of this sudden loss of candidates, the filter setting K is not as direct in its influence on the final EP set size as expected from the results of the medium-scale network. In addition, this results in the memory demand being relatively high for the EP output. The evolution of the memory demand is also tracked for the different filter settings and shown in the Supplementary Material.

## 4 Discussion

### 4.1 Benchmarking with other methods

The combination of the filtering approach with the candidate narrowing based on tree traversal leads to a well performing partial enumeration method of extreme rays. The filter setting helps scaling the enumeration problem, even though it has less direct control over the number of elementarity tests being carried out when compared to the methods described in Mores et al. (2025). Compared to similar existing methods, our approach achieves notable gains in computational efficiency. The widely used efmtool package, designed for full enumeration, includes optimizations that are not applicable to partial, filtering-based methods. Nevertheless, tree-based CBA outperforms it in speed even for full enumeration. This can be attributed to the more efficient elementarity testing available to CBA, which is not applicable to the NSA-based efmtool.

For the medium-scale network, the evolution of the number of rows in T is gradual, increasing the need for RAM towards the end of the algorithm. However, still only 2.48 GB RAM due to storing T as a sparse matrix and the tree evaluation only needing storage of union patterns, which are binary. Compared to efmtool, which uses 4.57 GB RAM, much less memory is used. This could be attributed to the way it does its rank calculations. The rank update method (Terzer and Stelling, 2008) relies on previously decomposed submatrices of S, which have to be saved during the tree traversal step. This saves a significant amount of computation, but increases the RAM usage, needing to store many decomposed submatrices. Reducing the RAM usage without loss of performance is one of the main targets to make double description-based approaches relevant to genome-scale metabolic networks.

### 4.2 Applicability to genome-scale metabolic networks

As was discussed previously, CBA is still very stochastic. In many cases, a small set of EPs is calculated as vital connections were lost during the algorithm through filtering. In the medium-scale network, a similar behaviour can be observed for low filter settings. However, for larger filter settings, this behaviour disappeared (Figure 7). For the results of the genome-scale network, the higher filter settings could not be reached with a normal desktop computer as the RAM demands became too high. Further investigation on data usage throughout the algorithm shows that the largest size of T happens before the final iterations for the genome-scale network (Figure 7), which is in contrast to the results of the medium-scale network. After this initial surge in T size, which can be attributed to the combinatorial explosion of candidates (Machado et al., 2012), the number of rows does not recover fully afterwards. It seems that after the initial peak, the main filtering effect has taken place and the T size does not recover with combinatorial explosion of candidates in later iterations. The expectation, based on medium-scale results, was that the peak in size would be relatively stable due to a consistent filter across the final iterations. However, it seems that genome-scale networks concentrate their complexity in these earlier iterations and then later iterations show a consistent de-growth of T .

There are some ways to deal with this explosion of candidates, which could be adapted to fit the filtering approach. Flux modules (Reimers et al., 2015; Müller and Bockmayr, 2014) help analyze and break down complex metabolic networks, which would reduce the genome-scale network and split it into modules corresponding to medium-scale subnetworks. This methodology has already been applied successfully to generate the optimal-yield EFMs, and thus relying heavily on the assumptions of FBA. Alternatively, an approach similar to demand-based subnetwork splitting as described in Hunt et al. (2014) could be adapted for CBA. Here, subnetworks are split based on forced inclusion or removal of certain reactions. For NSA, this is doable since each iteration is based on a specific reaction. For CBA, the demand-based splitting could be adapted, but would require to shift towards metabolite instead of reactions as they are the focus of the iterations in CBA. A first run of the algorithm with low filter setting could identify where most of the complexity is located, targeting certain metabolites for splitting. Each subnetwork could then be enumerated with higher filter settings to get quality samples.

When only considering the results with mainly type I EPs, tree-based CBA shows significant growth in terms of output versus filter setting K (Figure 5). When also considering the computation time for these runs (Figure 6), the novel approach seems to scale well with increasing problem sizes. If remedies are found to the evolution of T size, much higher computational efficiency could be obtained, which could bring double description methods into relevance for genome-scale applications.

## 5 Conclusion

The effective generation of extreme rays for genome-scale metabolic network is an important issue since they provide valuable insights into the metabolism of the cell. Their sheer volume severely limits the use of methods based on double description, which is used most frequently in the older studies with smaller networks due to its ability to deal with the high degree of degeneracy present in metabolic networks. Initial approaches (Mores et al., 2025; Machado et al., 2012) defined a way of only generating subsets of the extreme rays, thus limiting the memory issues caused by the vast number of extreme rays for genome-scale networks. However, they still had limitations in terms of computational efficiency. This work showcases a novel approach, integrating the tree-based candidate narrowing step from full enumeration methods such as efmtool into the sampling-based framework. To facilitate this integration, a new approach to filtering the candidates is introduced during the candidate narrowing step.

The new filtering approach showed consistent scaling in the medium-scale benchmark, while genome-scale performance still showed large stochastic behaviour and was memory-limited. The inclusion of candidate narrowing vastly improves the computational efficiency of the CBA method, which allows the exploitation of its more efficient elementarity test in the candidate narrowing step. For the medium-scale network, the speedup for the largest EP sets is more than 30 times. When compared to the popular efmtool package for the e_coli_core network under the benchmark conditions, our approach is twice as fast, despite efmtool’s advantages in full enumeration studies. The combinatorial explosion of candidates is found to still pose problems for genome-scale metabolic networks, even with the sampling of candidates through the iterations. The medium-scale case study shows a growth of candidates across the iterations, which levels out when the filter activates. However, for the genome-scale case study, the behaviour is quite different. The growth of candidates also happens across the iterations until filter activation, but afterwards there is a very large reduction in the last iterations. This limits the EP output, frequently only returning a small set of the less important type II and III EPs. The scalability of the problem does seem to be valid using filter setting K when the results with mainly type II and III EPs are omitted. But, the issue of the large reduction in candidates near the end of the algorithm should be addressed as to reduce the memory bottleneck which currently severely limits the extreme ray analysis in genome-scale metabolic networks.

## Data Availability

The tree-based Extreme Pathways generator used throughout this work is available online on GitLab KU Leuven and can be installed at the relevant commit with pip install git+ https://gitlab.kuleuven.be/biotec-plus/epgenerator. In the repository, data matrices are available in a supplementary folder, which allow the figures in this manuscript to be reproduced.
