# Supplementary material for: Partial enumeration of extreme rays in metabolic networks using bit pattern trees
Source: Front Bioinform. 2026 Sep 1;6:1892684. doi: 10.3389/fbinf.2026.1892684 (PMC13574982; doi:10.3389/fbinf.2026.1892684)
Supplement: Supplementary file 1 [file DataSheet1.pdf]

Supplementary Material

Wannes Mores

In this document, further analysis of the stochastic extreme pathway generation method is provided. After checking the accuracy of the method through comparison with an established method, the method is evaluated in terms of its bias towards pathway length and its memory usage throughout the iterations.

1 Validation of the pathway set

To validate the accuracy of the generated set of EPs with tree-based CBA, the full EP set is compared to the EP set generated with the popular *efmtool* package. After the initial run, tree-based CBA generates 100313 EPs, while *efmtool* generates a total of 100320 EPs. However, after removal of pathways only consisting of an internal cycle, both methods remain at 100273 pathways.

As introduced by Gagneur & Klamt (2004), it is sufficient to represent an EFM or EP through a binary representation, indicating reaction participation per pathway. As there are likely differences in the coefficients of the reactions in pathways from both methods, a binary representation helps for direct comparison between the methods. For each reaction, the number of pathways it is active in is counted and shown in Table 1. Reversible reactions are split into their forward and backward components.

Table 1: Reaction participation for full extreme pathway set

Reaction ID	efmtool	tree-based CBA
PFK	98637	98637
PFL	75009	75009
PGI	88625	88625
PGK	0	0
PGL	74606	74606
ACALD	0	0
AKGt2r	0	0
PGM	0	0
PIt2r	83601	83601
ALCD2x	0	0
ACALDt	0	0
ACKr	0	0

Table 1: Reaction participation for full extreme pathway set

Reaction ID	efmtool	tree-based CBA
PPC	92556	92556
ACONTa	96975	96975
ACONTb	96975	96975
ATPM	5650	5650
PPCK	5949	5949
ACt2r	0	0
PPS	11283	11283
ADK1	11283	11283
AKGDH	37707	37707
ATPS4r	21310	21310
PTAr	15909	15909
PYK	35714	35714
BIOMASS_Ecoli_core_w_GAM	83601	83601
PYRt2	0	0
CO2t	11424	11424
RPE	36036	36036
CS	96975	96975
RPI	0	0
SUCCt2_2	16338	16338
CYTBD	84170	84170
D_LACt2	0	0
ENO	100262	100262
SUCCt3	57367	57367
ETOHt2r	0	0
SUCDi	39542	39542
SUCOAS	0	0
TALA	54968	54968
THD2	26462	26462
TKT1	54968	54968
TKT2	17303	17303
TPI	98491	98491
EX_ac_e	15909	15909
EX_acald_e	15023	15023
EX_akg_e	25418	25418
EX_co2_e	56197	56197
EX_etoh_e	48770	48770
EX_for_e	75009	75009
EX_fru_e	0	0
EX_fum_e	0	0
EX_glc__D_e	100273	100273
EX_gln__L_e	0	0
EX_glu__L_e	13419	13419

Table 1: Reaction participation for full extreme pathway set

Reaction ID	efmtool	tree-based CBA
EX_h_e	98715	98715
EX_h2o_e	72255	72255
EX_lac__D_e	14416	14416
EX_mal__L_e	0	0
EX_nh4_e	86619	86619
EX_o2_e	84170	84170
EX_pi_e	83601	83601
EX_pyr_e	13802	13802
EX_succ_e	48602	48602
FBA	98491	98491
FBP	5752	5752
FORt2	16338	16338
FORt	80733	80733
FRD7	24192	24192
FRUpts2	0	0
FUM	39542	39542
FUMt2_2	0	0
G6PDH2r	74606	74606
GAPD	100262	100262
GLCpts	100273	100273
GLNS	89451	89451
GLNabc	0	0
GLUDy	3630	3630
GLUN	5650	5650
GLUSy	44615	44615
GLUt2r	1	1
GND	74606	74606
H2Ot	6873	6873
ICDHyr	92208	92208
ICL	42246	42246
LDH_D	1	1
MALS	42246	42246
MALt2_2	0	0
MDH	16706	16706
ME1	29868	29868
ME2	36188	36188
NADH16	88046	88046
NADTRHD	31023	31023
NH4t	86619	86619
O2t	84170	84170
PDH	39579	39579
PGI_reverse	2265	2265

Table 1: Reaction participation for full extreme pathway set

Reaction ID	efmtool	tree-based CBA
PGK_reverse	100262	100262
ACALD_reverse	59014	59014
AKGt2r_reverse	25418	25418
PGM_reverse	100262	100262
PIt2r_reverse	0	0
ALCD2x_reverse	48770	48770
ACALDt_reverse	15023	15023
ACKr_reverse	15909	15909
ACONTa_reverse	0	0
ACONTb_reverse	0	0
ACt2r_reverse	15909	15909
ADK1_reverse	0	0
ATPS4r_reverse	15111	15111
PTAr_reverse	0	0
PYRt2_reverse	13802	13802
CO2t_reverse	44773	44773
RPE_reverse	38268	38268
RPI_reverse	93108	93108
D_LACt2_reverse	14416	14416
ENO_reverse	0	0
ETOHt2r_reverse	48770	48770
SUCOAS_reverse	37707	37707
TALA_reverse	18903	18903
TKT1_reverse	18903	18903
TKT2_reverse	57151	57151
TPI_reverse	105	105
EX_co2_e_reverse	0	0
EX_glc__D_e_reverse	0	0
EX_h_e_reverse	0	0
EX_h2o_e_reverse	0	0
EX_nh4_e_reverse	0	0
EX_o2_e_reverse	0	0
EX_pi_e_reverse	0	0
FBA_reverse	105	105
FUM_reverse	24192	24192
G6PDH2r_reverse	0	0
GAPD_reverse	0	0
GLUDy_reverse	44361	44361
GLUt2r_reverse	13419	13419
H2Ot_reverse	65382	65382
ICDHyr_reverse	0	0
LDH_D_reverse	14416	14416

Table 1: Reaction participation for full extreme pathway set

Reaction ID	efmtool	tree-based CBA
MDH_reverse	43283	43283
NH4t_reverse	0	0
O2t_reverse	0	0

2 Pathway length analysis

Filtering based approaches to generating extreme rays have shown a bias to shorter pathways in the past (Machado et al., 2012). To investigate this phenomenon for the tree-based CBA method, a range of filter settings were used with 10 repetitions per filter setting. The pathway lengths are shown as a relative frequency in the histograms in Figure S1. For comparison, the histogram of the full set is added to each plot.

For the low filter settings, there is a definite bias towards the shorter pathways. This bias can be attributed to the shorter pathways needing to pass less filtering compared to longer pathways. For example, pathways that have only 3 intermediary metabolites will only need to survive the filtering in 3 iterations of the CBA method as each iteration corresponds to the balancing of a single internal metabolite. For filter settings of 10^5 and onwards, the distributions are matching a lot more closely. If the number of EPs is taken into account, a relatively good representation is found for small sizes. For example, filter setting 10^5 results in a decent match, while having only a couple of thousand EPs.

3 Memory usage during the tree-based CBA method

As mentioned in the manuscript, the novel method allows the scaling of the problem such that it can be run with reasonable memory usage. However, memory still proves to be an issue as the EP output is relatively low for the peak memory usage. In the manuscript, the reason behind the lower output is shown through the evolution of the number of rows in the Tableau $\mathbf{T}$. In addition, the memory demand of the method is tracked and plotted in this section in Figure **??**. For filter settings over $K = 10^6$, the memory demand exceeded the maximum of 16 GB, making 10^6 the largest feasible filter setting. In contrast to the number of rows discussed in the manuscript, memory usage does not appear to decrease in later iterations. This behavior is likely due to Python's memory management: although objects from previous iterations may be deallocated, the memory is often retained by the Python allocator rather than being returned to the operating system, causing the process's memory footprint to remain high.

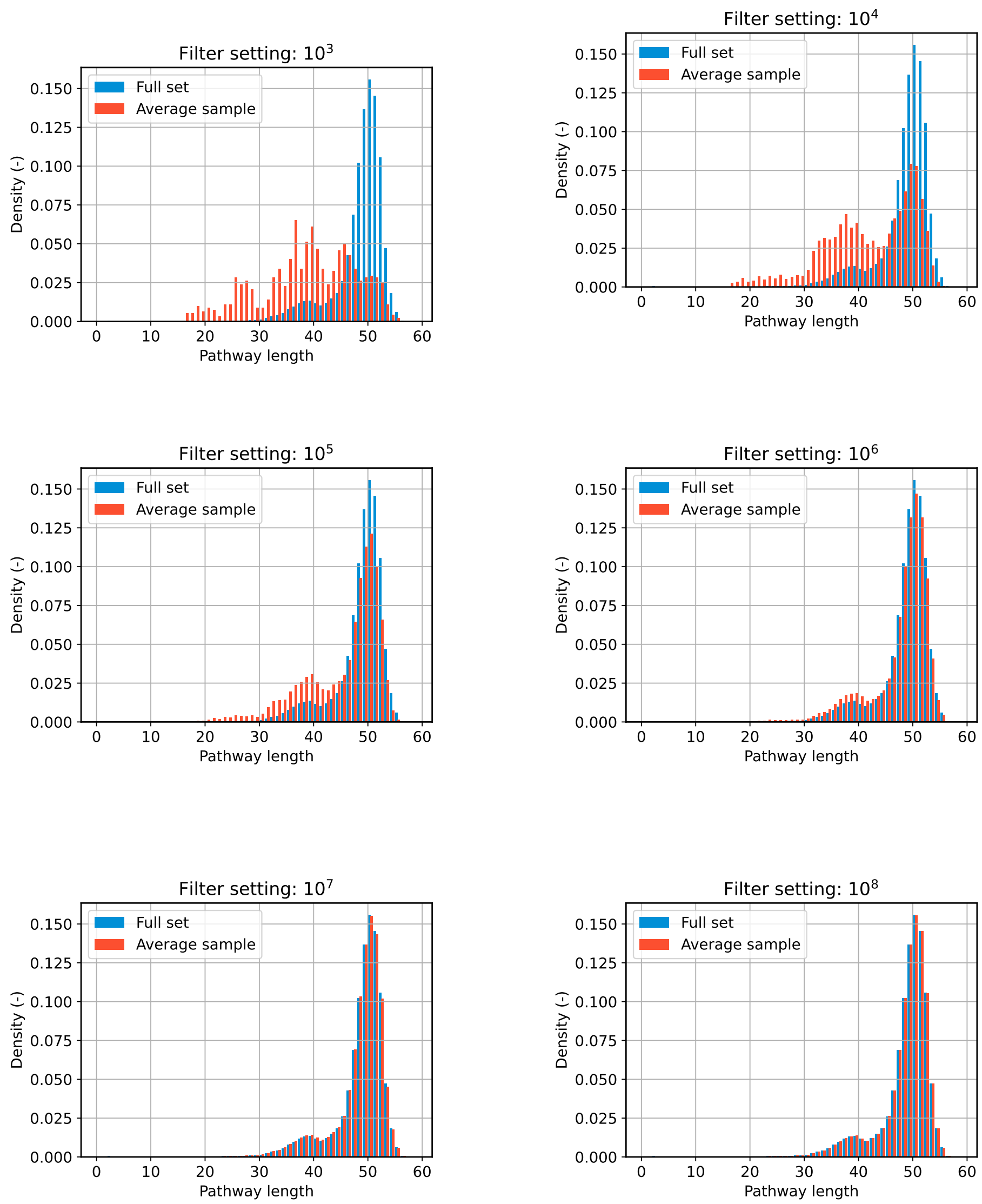

Figure S1: Comparison of the pathway length distributions generated by tree-based CBA. Per filter setting, the pathway lengths of 10 samples are averaged and compared to the distribution of pathway lengths for the full set.

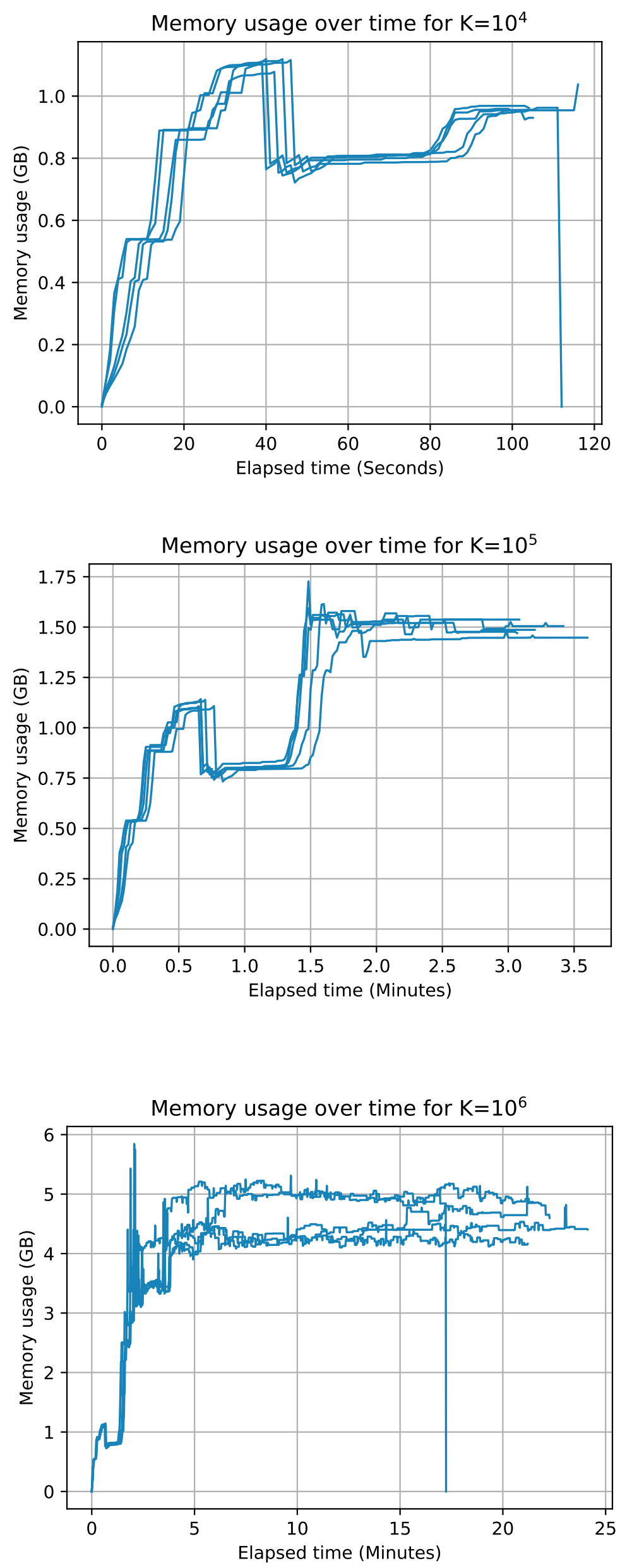

Figure S2: Memory usage evolution for the tree-based CBA at different filter settings. For a filter setting of $K = 10^7$, memory usage exceeded the maximum of 16 GB and is therefore not included.

3.1 GEM summary statistics

For the second case study, the proportion of pathway types was also evaluated to distinguish the runs. 8 runs were done per filter setting to check the evolution of pathway proportions. In the table below, summary statistics along with Type I pathway dominance are shown for the different filter settings. These values correspond to Figures 5-6 in the original paper. For more detailed information, a raw datafile in CSV format is uploaded to the repository.

Table 2: Summary statistics for different input sizes.

Filter	Type I < 80%	Type I > 80%	Average Type I %	Median EP Output	Median Time	IQR of EP output
10^2	8	0	15.08	110.0	17.953	102.25–133.25
$10^{2.25}$	8	0	20.27	112.5	18.163	103.75–174.25
$10^{2.50}$	8	0	41.07	198.5	18.533	144.25–251.50
$10^{2.75}$	7	1	54.38	286.0	18.938	170.00–444.00
10^3	7	1	50.83	222.0	19.723	171.50–361.25
$10^{3.25}$	5	3	60.51	316.0	20.826	223.50–766.00
$10^{3.50}$	4	4	63.59	445.5	23.036	241.25–736.25
$10^{3.75}$	6	2	48.56	230.0	25.673	135.00–638.50
10^4	7	1	47.36	206.5	32.152	149.00–322.25
$10^{4.25}$	8	0	49.98	296.5	43.383	146.25–412.25
$10^{4.50}$	5	3	56.52	235.0	62.930	161.75–646.00
$10^{4.75}$	6	2	45.64	183.5	118.459	131.25–525.25
10^5	6	2	50.99	220.5	197.447	149.75–526.75
$10^{5.25}$	7	1	49.70	210.0	338.184	148.75–404.50
$10^{5.50}$	7	1	28.96	137.5	667.527	124.75–165.25
$10^{5.75}$	7	1	49.36	252.5	1299.063	193.25–308.75
10^6	5	3	54.19	236.0	2801.650	147.75–2948.25

References

Gagneur, J., & Klamt, S. (2004). Computation of elementary modes: a unifying framework and the new binary approach. *BMC bioinformatics*, *5*, 175.

Machado, D., Soons, Z., Patil, K. R., Ferreira, E. C., & Rocha, I. (2012). Random sampling of elementary flux modes in large-scale metabolic networks. *Bioinformatics*, *28*, i515–i521.
